# Risk Assessment of Pectenotoxins in New Zealand Bivalve Molluscan Shellfish, 2009–2019

**DOI:** 10.3390/toxins12120776

**Published:** 2020-12-06

**Authors:** Michael J. Boundy, D Tim Harwood, Andreas Kiermeier, Cath McLeod, Jeane Nicolas, Sarah Finch

**Affiliations:** 1 Cawthron Institute, 98 Halifax Street East, Nelson 7010, New Zealand; tim.harwood@cawthron.org.nz; 2 New Zealand Food Safety Science and Research Centre, Massey University, Private Bag 11-222, Palmerston North 4442, New Zealand; C.McLeod@massey.ac.nz; 3 Statistical Process Improvement Consulting and Training PTY Ltd., Gumeracha, SA 5233, Australia; andreas.kiermeier@gmail.com; 4 Ministry for Primary Industries–Manatu Ahu Matua, P.O. Box 2526, Wellington 6140, New Zealand; Jeane.Nicolas@mpi.govt.nz; 5 AgResearch Limited, Ruakura Research Centre, Private Bag 3123, Hamilton 3240, New Zealand; sarah.finch@agresearch.co.nz

**Keywords:** Pectenotoxin, Exposure, Risk assessment, Diarrhetic shellfish poisoning

## Abstract

Pectenotoxins (PTXs) are produced by *Dinophysis* spp., along with okadaic acid, dinophysistoxin 1, and dinophysistoxin 2. The okadaic acid group toxins cause diarrhetic shellfish poisoning (DSP), so are therefore regulated. New Zealand currently includes pectenotoxins within the DSP regulations. To determine the impact of this decision, shellfish biotoxin data collected between 2009 and 2019 were examined. They showed that 85 samples exceeded the DSP regulatory limit (0.45%) and that excluding pectenotoxins would have reduced this by 10% to 76 samples. The incidence (1.3%) and maximum concentrations of pectenotoxins (0.079 mg/kg) were also found to be low, well below the current European Food Safety Authority (EFSA) safe limit of 0.12 mg/kg. Inclusion within the DSP regulations is scientifically flawed, as pectenotoxins and okadaic acid have a different mechanism of action, meaning that their toxicities are not additive, which is the fundamental principle of grouping toxins. Furthermore, evaluation of the available toxicity data suggests that pectenotoxins have very low oral toxicity, with recent studies showing no oral toxicity in mice dosed with the PTX analogue PTX2 at 5000 µg/kg. No known human illnesses have been reported due to exposure to pectenotoxins in shellfish, a fact which combined with the toxicity data indicates that they pose negligible risk to humans. Regulatory policies should be commensurate with the level of risk, thus deregulation of PTXs ought to be considered, a stance already adopted by some countries.

## 1. Introduction

Pectenotoxins (PTXs) are produced by *Dinophysis* spp. [1], and during blooms of this microalgal species, filter feeding bivalve mollusks can accumulate the microalgae in their digestive glands and absorb lipophilic compounds they produce into the shellfish flesh. In addition to PTXs, *Dinophysis* spp. also produces okadaic acid group toxins; okadaic acid (OA), dinophysistoxin 1 (DTX1), and dinophysistoxin 2 (DTX2). Toxins from the OA group have been known to cause human illness since the late 1970s [2], inducing a syndrome called diarrhetic shellfish poisoning (DSP), which is dominated by the symptom of diarrhea. To minimize the incidence of this illness, regulatory limits have been set for OA group toxins found in shellfish. Historically, due to the co-production and co-occurrence of PTXs and okadaic acid group toxins by *Dinophysis* spp., PTXs have been included in DSP regulation, and this is still the case in New Zealand.

When monitoring methods moved away from the traditional mouse bioassay to analytical analysis, it was discovered that the PTXs consisted of a large array of 20 related analogues, although only PTX1 and PTX2 are included in the DSP regulation, with PTX1 not routinely monitored due to unavailability of suitable reference material. Shellfish samples that contain toxin concentrations above the maximum permissible level for DSP result in the closure of the shellfish harvesting area until the toxin levels have returned to safe concentrations. Little is known about the distribution of PTXs in New Zealand shellfish or the concentrations present in shellfish. The Ministry for Primary Industries and the New Zealand shellfish industry have tested for marine biotoxins in bivalve molluscan shellfish for many years, yielding a large set of data. The presence of PTXs in shellfish is typically monitored using liquid chromatograph-tandem mass spectrometry (LC-MS/MS) [3,4]. Using this approach, a range of PTXs have been reported, including the PTX analogue PTX2, and the non-regulated metabolites PTX2SA and 7-epi-PTX2SA, which are collectively reported as pectenotoxin 2 seco acids (PTX2SAs). When the method was first developed, PTX1, PTX11, and PTX6 were not routinely monitored due to instrument limitations [4]. However, with advancements in instrumentation, these three additional analogues are now acquired simultaneously by the LC-MS/MS method used for regulatory monitoring in New Zealand without impacting method performance. However, while these congeners have been added to the acquisition method, they are not included in the routine processing and quantitation due to the additional time and cost required.

In this study, to fill knowledge gaps surrounding PTXs in New Zealand shellfish, information gathered from 2009–2019 was used to collate prevalence data for OA, DTX1, and DTX2 (after hydrolysis), as well as PTX2 and its seco acids over the 10-year period. In addition, for selected bloom events, the concentrations of the PTX analogues, PTX1, PTX11, and PTX6, were obtained by manually reprocessing historical LC-MS/MS data acquired in order to retrospectively determine PTX profiles within New Zealand shellfish. Using these data, the impact of including PTXs in the DSP class of toxins was evaluated. To be able to conduct an exposure assessment for PTXs, the concentrations in shellfish must be combined with the quantity eaten by the consumer (meal sizes). Unfortunately, most consumption surveys are targeted to obtain data on consumption over time, which is best suited to chronic toxicity risk assessments. Because consumption surveys are often summarized as the “average amount of a food consumed over the survey period”, it is usually impossible to discern the frequency and amount per serving. Knowing only the average amount consumed (e.g., 50 g/day) does not provide information on whether a consumer eats consistent portions daily throughout the week, or whether larger portions (e.g., 175 g/meal) are consumed on average a couple of times per week. In the Oct 2008–Oct 2009 New Zealand Adult Nutrition Survey [5], a 24-h recall of 4721 adults aged 15+, including 1040 Maori and 757 Pacific peoples, was used. It was not stated if people consumed more than one type of the seafood listed, so a total mollusk consumption could not be determined. The highest 97.5 percentile portion size across the shellfish species was 268 g (paua), followed by 256 g for mussels. While insufficient data are available to create a robust meal size distribution for risk modeling, an approximation can be made using simulations, such as a triangular distribution [6]. 

To conduct a risk assessment of the PTXs, information on not only exposure, but also toxicity of the compounds is required. There are many reports of intraperitoneal injection (i.p.) toxicity of PTXs in mice. However, information on the feeding method, strain, and sex of mice is not documented in most of the available publications, which makes the interpretation and accurate comparison of the data difficult. It is clear that PTX1, PTX2, PTX3, and PTX11 are of similar toxicity by i.p. administration, with lethal doses of between 219 and 411 µg/kg; PTX4 and PTX6 appear to be slightly less toxic, with lethal doses of 770 and 500 µg/kg, respectively, and PTX7, PTX8, PTX9, PTX2SA, and 7-epi-PTX2SA are of low toxicity, with no mouse deaths observed even at a dose rate of 5000 µg/kg [1,7,8,9,10,11,12]. In comparison to the i.p. route of administration, there have been few studies conducted to investigate the acute oral toxicity of PTXs. The first report was by Ishige et al. in 1988 [13], which stated that the lowest observed adverse effect level (LOAEL) was 250 µg/kg based on a single mouse dosed by gavage with PTX2 of unspecified purity. The effects observed in the study involved fluid accumulation in the intestine and damage to intestinal villi of the mouse. Using this figure, the EFSA CONTAM Panel derived an acute reference dose (ARfD) of 0.8 µg PTX2 equivalents/kg b.w., and derived a safe level of 0.12 mg/kg in shellfish flesh based on a 400 g large portion size [6]. Although focused on yessotoxins, a study in the 1990s [14] reported what appeared to be the oral acute toxicity of PTX2. In this study, the oral toxicity of PTX2 was reported to be similar to the toxicity by i.p. injection. In contrast, the study by Miles et al. [10] showed no signs of toxicity in any of the five mice dosed with PTX2 at a dose rate of 5000 µg/kg using well-characterized material. The acute oral toxicity of PTX2SA [10] and PTX11 [12] was found to be equally low, with no signs of toxicity observed in any of the five mice dosed with either compound at a dose rate of 5000 µg/kg. The severe diarrhea in mice attributed to PTX2 in the earlier study by Ishige may have been due to contamination of the sample with an okadaic acid derivative, which is co-extracted with PTX2 [10]. The question of the toxicity of PTXs is essential in conducting a risk assessment, and underpins whether they should be regulated. Furthermore, the validity of including PTXs with the OA group toxins is investigated. Various areas of the world handle the regulation of PTXs differently, so, in this study, we will review the available literature, which is often conflicting, and present a rationale for the interpretation of the data.

## 2. Results

### 2.1. Distribution of PTXs in New Zealand

#### 2.1.1. Spatial Distribution of PTXs

**Figure 1:**
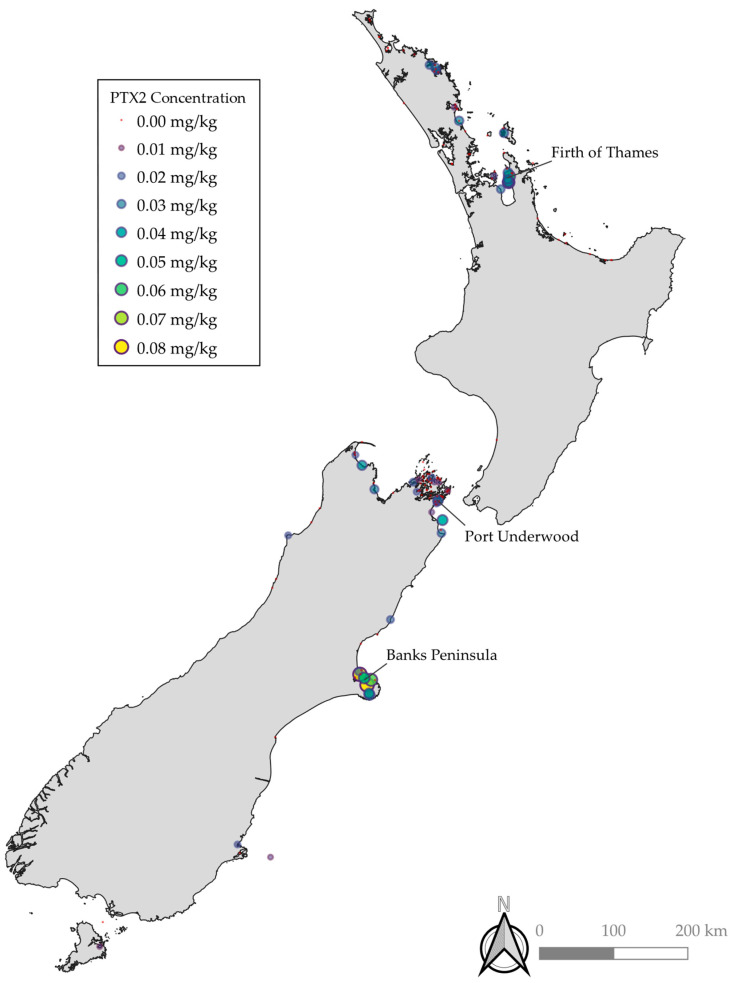
Maximum concentrations of PTX2 at sampling locations throughout New Zealand. Marker color and size are on a continuous scale.

PTX2 and *Dinophysis* spp. were both detected throughout the country with notably elevated concentrations and occurrence observed in Banks Peninsula, the Firth of Thames, and Port Underwood (Figure 1). Relative concentrations of PTX2, PTX2SAs, and DSP were similar across the different regions, with typically PTX2SAs > DSP > PTX2 (DSP toxins = OA, DTX1, and DTX2). However, in some bloom events, there were notably relatively less PTX2 and PTX2SAs compared to DSP toxins. These may be due to blooms of other species, such as *Prorocentrum* spp., which are known to produce OA group toxins but not PTXs. Benthic species, such as *Prorocentrum* spp., do not reliably get detected with routine phytoplankton monitoring. The locations of *Dinophysis* spp. detection were similarly consistent with the observations of PTX2, PTX2SAs, and DSP toxins. However, no phytoplankton samples from the West Coast have been tested where a PTX2/DSP bloom was detected due to the difficulty in obtaining samples caused by inaccessible terrain and weather. The concentrations of *Dinophysis* spp. cell counts did not correlate to detections of PTX2, PTX2SAs, and DSP toxins, with some higher cell counts not resulting in higher concentrations of PTX2, PTX2SAs, and DSP. This is likely due to differences in the production of toxins between algal species, and potentially non-producing strains.

#### 2.1.2. Temporal Distribution of PTXs

**Figure 2:**
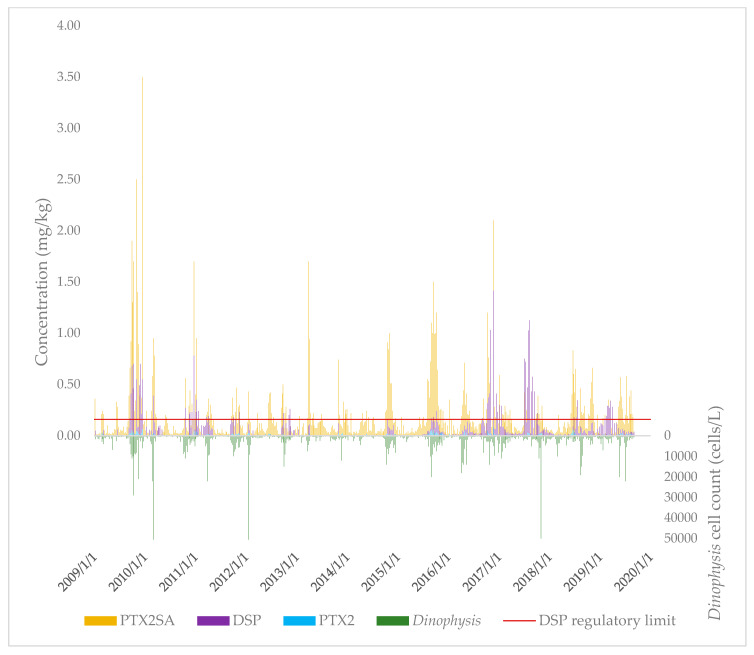
Concentrations of PTX2, PTX2SAs, DSP, and *Dinophysis* spp. throughout New Zealand over the 2009–2019 period.

The concentrations of PTX2, PTX2SAs (sum of PTX2SA and 7-epi-PTX2SA), and DSP toxins in New Zealand (independent of sample site) over the 2009–2019 period were plotted over time, together with the *Dinophysis* spp. cell concentrations (Figure 2).

Results were grouped by year in order to assess potential changes in occurrence over the 2009–2019 period. PTX2 results are summarized in Table 1.

Both 2009 and 2015 showed elevated bloom occurrence, with 3.3–3.5% of samples having detectable PTX2 compared to the other years, where only 0.6–1.4% of the samples had detectable PTX2.

Results were grouped by month in order to assess potential seasonality, with PTX results summarized in Table 2. Detections of PTX2 were observed in all months of the year, with the largest number of detections in September–October, and maximum concentrations observed in November–December.

#### 2.1.3. Species Distribution of PTX2

Sample results were sorted by type of shellfish, and results for PTX2 are summarized in Table 3. The data available from the laboratory information management system (LIMS) database only identified species by a common name. The most commonly tested type of shellfish was green-lipped mussels (*Perna canaliculus*, 84%), followed by Pacific oyster (*Crassostrea gigas*, 6%), clams (unspecified, 5%), scallops (*Pecten novaezealandiae*, 2%), and dredge oyster (*Ostrea chilensis*, 1%). Small numbers of other shellfish species (< 1% each) were also analyzed. Blue mussels (*Mytilus edulis*) had the highest detection rate for any shellfish type (12.5%). This observation is likely impacted by sampling bias, as blue mussels are typically not analyzed as part of routine monitoring in New Zealand, and are instead taken from areas in response to a bloom event.

#### 2.1.4. Pectenotoxin Profiles

**Figure 3:**
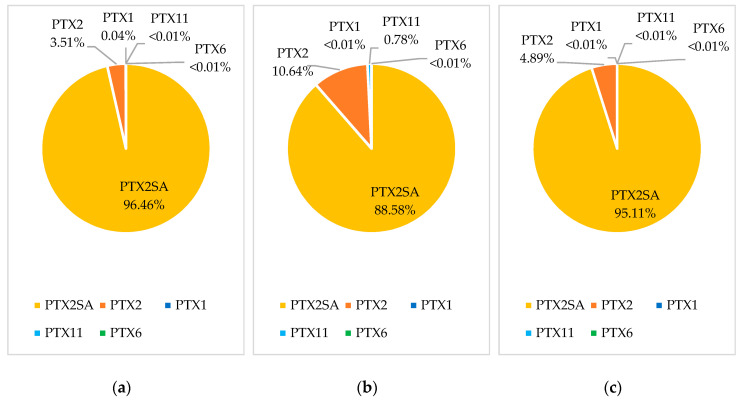
Pectenotoxin profiles based on the 97.5 percentile concentrations for PTX analogues in the Coromandel 2015 bloom for: (**a**) green-lipped mussels; (**b**) Pacific oyster; (**c**) scallops.

Samples from five bloom events were reprocessed to quantify PTX1, PTX11, and PTX6. Multiple reaction monitoring (MRM) transitions for these analogues are acquired using the LC-MS/MS method of analysis, although they are not routinely processed [4]. The three blooms with the highest observed concentration of PTX2 were selected for reprocessing, as well as the two highest concentration blooms from areas where Pacific oyster and scallops were most commonly sampled. There were no detections of PTX1, PTX11, or PTX6 above the 0.01 mg/kg reporting limit in any of the 389 reprocessed samples. Trace detections were observed for PTX1 and PTX11 in some samples, and PTX6 was not detected in any samples. As only trace detections were observed, profiles were assessed including all trace detections and including those below the quantitation and reporting limits. A bloom event in the Coromandel region in 2015 affected the largest number of sites, species, and samples, and provided the richest dataset of the bloom events observed in the 2009–2019 period. PTX profiles from this 2015 bloom event are shown for green-lipped mussels (n = 182), Pacific oysters (n = 10), and scallops (n = 3) in Figure 3.

Green-lipped mussels showed a trace detection of PTX1, with an average of 1.1% of the concentration of PTX2. The PTX1 detections showed a similar accumulation and depuration trend to PTX2. PTX1 and PTX6 have previously been reported to form via metabolism in Japanese scallops (*M. yessoensis*) [7,15]. However, PTX1 is only observed at a very low abundance, in contrast to that observed in *M. yessoensis*. Pacific oysters showed a trace detection of PTX11, with an average of 13.1% of the concentration of PTX2. As there were only three Pacific oyster samples analyzed during the bloom that showed trace levels of PTX11, there was not enough information to observe an accumulation and depuration trend. Pacific oysters also showed a lower abundance of PTX2SAs, and this could be due to different binding of the compounds within the flesh, lower levels of PTX2 metabolism than in green-lipped mussels, or due to competing metabolism to form other congeners, such as PTX11. Scallops contained no detectable levels of either PTX1 or PTX11.

#### 2.1.5. Impact of PTXs Contribution to DSP Levels

**Figure 4:**
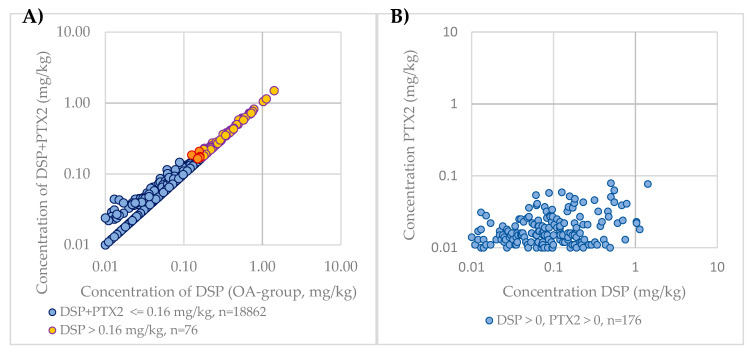
(**A**) Comparison of PTX2 contribution to DSP regulation in New Zealand over the 2009–2019 period on a logarithmic scale; (**B**) Comparison of PTX2 concentrations to DSP concentrations over the 2009–2019 period on a logarithmic scale.

In New Zealand, PTXs are currently included in the DSP regulation. To compare the impact of the inclusion of PTXs in regulatory monitoring, the DSP (excluding PTX2) concentration and sum of DSP and PTX2 concentrations were calculated for each sample. As PTX2 is the only PTX group congener that is routinely monitored and is the most dominant congener, apart from the non-regulated seco acids, PTX2 was used as a surrogate for PTXs. These results were then compared against the current regulatory limit, with results shown in Figure 4A. As the OA group toxin concentration increases with samples where PTX2 is detected, the DSP + PTX2 concentration becomes closer to the DSP concentration, as the PTX2 concentration does not proportionately increase with the DSP concentrations (Figure 4B).

Of the 18,947 samples analyzed, a total of 76 samples were above the regulatory limit for DSP (0.4%). An additional nine samples were considered above the DSP regulatory limit with the inclusion of PTX2. Of the nine samples in the 2009–2019 period that were pushed above the regulatory limit by including the PTX2 concentration, three of the OA group concentrations were at the regulatory limit (0.16 mg/kg), five were at 0.15 mg/kg, and one at 0.13 mg/kg; PTX2 concentrations for these samples were between 0.01 and 0.059. There were 75 samples that contained reportable levels of PTX2 and no reportable OA group toxins, with 90% of these samples analyzed prior to July 2015, when the limit of reporting for the OA group analogues was 0.05 mg/kg rather than 0.01 mg/kg due to use of a less sensitive instrument. 

### 2.2. Risk Assessment

#### 2.2.1. Deterministic Risk Assessment

Three portion sizes were used to assess exposure to PTX2: 100 g, the standard portion size [16]; 268 g, the highest 97.5 percentile portion size of shellfish species for New Zealand consumers; and 400 g, the large portion size adopted by EFSA for risk assessment [6]. The exposure for a consumer of a large (400 g) portion of shellfish meat contaminated with the maximum concentration of PTX2 observed from all samples over the 2009–2019 period is 0.53 µg PTX2/kg b.w. (Table 4), which is still less than the ARfD of 0.8 µg PTX2/kg b.w. proposed by EFSA [6]. A 60 kg person would have to consume approximately 608 g of shellfish at 0.079 mg PTX2/kg to reach this conservative ARfD.

The main components of the exposure assessment and risk characterization were the consumption amount of bivalve mollusks and the distribution of PTX2 concentrations in bivalve mollusks. A probabilistic estimate of dietary exposure to PTX2 was performed by a Monte Carlo simulation to generate the amount of PTX2 consumed in a sitting (adjusted by kg body weight). The focus of the risk assessment was on the acute exposure of the consumption of PTX2.

#### 2.2.2. Probabilistic Risk Assessment

The risk characterization is the comparison of the exposure distributions to the corresponding Health Based Guidance Value, which for PTX2 is the conservative ARfD of 0.8 μg/kg b.w. proposed by EFSA [6]. None of the 1,000,000 iterations in either of the models for the Monte Carlo simulations resulted in an exposure exceeding the ARfD (based on the maximum of 0.533 µg/kg b.w.). This is consistent with the absence of reported human illnesses due to exposure to PTXs.

## 3. Discussion

An examination of biotoxin data collected in New Zealand between 2009 and 2019 showed PTXs to be present throughout the country, in a range of shellfish species, with detections more frequent in September and October and maximum PTX2 concentrations observed in November (0.063 mg/kg) and December (0.079 mg/kg). However, the number of PTX2 detections was low, as demonstrated by the observation that only 3.3–3.5% of shellfish samples collected in the years with the highest number of detections (2009 and 2015) contained PTX2. The PTX profiles were examined in three shellfish species, which showed PTX2SA to be the dominant PTX compound (89–96%), followed by PTX2 (3.5–10.6%), PTX11 (0–0.78%), and PTX1 (0–0.04%); PTX6 was not detected in any of the shellfish samples.

Since New Zealand includes PTXs in the DSP regulation, in contrast to International Codex Standard 292-2008, the impact of PTX2 to DSP levels was investigated in this research. It was found to be minor. Over the 10 years of data examined, 76 shellfish samples were determined to be above the DSP regulatory limit (excluding PTX2) (0.4%), and only an additional nine samples (0.05%) were pushed over the regulatory limit by the inclusion of PTX2. When comparing the contribution of PTX2 to OA group toxins in shellfish where PTX2 was detected, there was a relatively higher contribution of PTX2 at lower concentrations of OA group toxins. This could be due to the metabolism of PTX2 to PTX2SA in New Zealand shellfish. As PTX2 and OA group toxins are accumulated by the shellfish, PTX2 is metabolized to PTX2SA over time, resulting in relatively lower PTX2 concentrations compared to OA group toxins as the bloom progresses and OA group toxin concentrations increases. From the deterministic risk assessment of PTX2, the highest concentration observed in shellfish over the 2009–2019 period would require a large 608 g portion size to be consumed in order to reach the conservative ARfD proposed by EFSA. With the probabilistic risk assessment of PTX2, there were no simulated cases exceeding this ARfD. 

The grouping of related toxins for the assessment of human exposure is essential, as toxicity is generally not due to one individual compound, but rather a mixture of related structural analogues. Since the mouse bioassay has been proven to be inaccurate and is considered by many countries to be unethical for routine screening, this is now handled by instrumental chemical analysis of shellfish samples for all known analogues of the DSP toxin class. Since analogues will have different toxicities, to translate this into an estimate of overall toxicity, the relative toxicities of the individual components must be applied. To determine toxicity equivalence factors (TEFs), toxicity data is considered with the following order of importance: data from human cases (outbreaks) > oral LD_50_ in animals > i.p. LD_50_ in animals > mouse bioassay and in vitro data [17]. The fundamental principle for grouping toxins is that they must have a shared mechanism, hence, their toxicities are additive [18]. This requirement is met for OA and the DTXs, as both are active on protein phosphatases. However, PTXs are inactive on protein phosphatases, and instead exert their effect by action on F-actin [19]. In our view, including the PTX group as part of the DSP regulation is therefore scientifically not justified. This position is consistent with the view expressed by numerous scientific opinions and FAO/WHO/IOC committees [6,16,18,20,21,22]. Despite these clear and numerous scientific opinions, some countries, including New Zealand, Canada, Chile, and the EU, currently include PTX2 in the DSP regulation, whereas other countries, including Australia, Japan, the United States of America, and Mexico, do not.

To provide an estimate of the acute risk of PTXs to human health, the most relevant parameter is the toxic dose by oral administration. The non-toxicity of PTX2 observed by Miles et al. [10] is at odds with the early study by Ishige et al. [13]. Another difference in the studies was that, in contrast to the early study, the one conducted in 2004 reported no diarrhea in mice dosed with PTX2. While diarrhea is a well-recognized symptom of the OA group toxins, whether PTXs induce diarrhea or not is a key point in assessing the validity of the Ishige et al. and Miles et al. toxicity assessments of PTX2. PTX1 has been shown to induce no diarrhea when injected into either suckling mice [23] or when administered by gavage [24]. Furthermore, using intestinal models, it has been shown that PTX1, unlike OA or the DTXs, caused no fluid accumulation in rabbit or mouse intestinal loops [24]. Since PTXs are co-extracted with the OA group toxins, and they are difficult to separate, it appears likely that the early report of PTX2 toxicity by gavage utilized material contaminated with an OA derivative, hence inducing diarrhea and giving an incorrect assessment of toxicity [10]. The other report of PTX2 inducing oral toxicity is the study by Ogino et al. [14], who found that the oral toxicity of PTX2 was similar to that generated by i.p. injection. However, the results reported are dubious, as no dose dependency was observed. The mortality recorded at a dose rate of 25 µg/kg (25%) was higher than that seen in mice dosed at both 100 µg/kg (0%) and 200 µg/kg (20%), while the mortality observed in mice dosed at 400 µg/kg (25%) was equal to that of the 25 µg/kg (25%) group, and lower than that recorded in mice dosed at 300 µg/kg (40%). It is difficult to account for this observed non-dose dependent mortality, but it should be noted that there can be a high incidence of gavage-associated deaths and that the administration technique can impact on the results [25,26,27]. The most robust study of PTX2 toxicity is therefore that of Miles et al. [10], which reported no signs of toxicity in mice dosed with 5000 µg/kg of well-characterized PTX2. PTX11, which has a similar i.p toxicity as PTX2, was equally non-toxic at an oral dose rate of 5000 µg/kg [12]. The major metabolic product of PTX2, PTX2SA, was also non-toxic orally at this dose rate [10].

Since EFSA based the ARfD on the oral toxicity reported in 1988, which we now believe to be incorrect, the ARfD should be reevaluated. The highest dose tested in the more recent toxicity studies was 5000 µg/kg, which induced no toxic effects and hence represents a no observed adverse effect level (NOAEL) rather than an LOAEL or LD_50_, which may be considerably higher. If this figure of 5000 µg/kg is used to calculate an ARfD, taking into account a 10-fold safety factor for a possible toxicity difference in species and a 10-fold safety factor for possible toxicity variation within species, an ARfD of 50 µg/kg is generated, over 60-fold higher than the ARfD proposed by EFSA. For a 60 kg standard adult human, applying the 400 g large portion meal size proposed by EFSA gives a level of PTX2 equivalents that would be considered safe of 7.5 mg PTX2 equivalents/kg mollusk flesh. This is approximately 100-fold higher than the maximum observed concentration of PTX2 in New Zealand shellfish over the 2009–2019 period.

There is a total lack of toxicity in mice dosed with PTX2, PTX11, or PTX2SA orally at a dose rate of 5000 µg/kg, and a lack of toxicity observed in mice dosed with PTX7, PTX8, PTX9, PTX2SA, and 7-epi-PTX2SA by i.p. at a dose rate of 5000 µg/kg. Although no oral sub-chronic toxicity data are available, the difference between the i.p. and oral toxicity seen for PTX2 and PTX11 can be explained on the basis of a low or lack of absorption of these compounds in the gastrointestinal tract. Consistent with this assumption, after an oral administration of a mixture of PTX2 and PTX2SA, the majority of the toxins remained within the gastrointestinal tract and were excreted in the feces [28]. On this basis, the risk of cumulative toxicity is very low, and the acute toxicity data would give a good estimation of the overall risk posed by PTXs. These data show no oral toxicity, even at very high dose rates, which is consistent with the total lack of any evidence implicating PTXs in human illness, a fact recognized in various EFSA and WHO documents [6,16,29]. A review of all available data therefore suggests that PTXs do not pose a health risk. This view is also shared by FAO/WHO/IOC panels, who have regularly discussed PTXs, with the consensus being that there is no recommendation to regulate this toxin class [16,18,20,22,30].

This study has shown that the contribution of the PTXs to the DSP group toxin concentration is small and that the risk to human health posed by the occurrence of PTXs in shellfish in New Zealand is negligible, with the probabilistic risk assessment showing no simulated cases that exceed even the current ARfD. A comparison of the mechanisms of action for PTXs and DSP group toxin classes show them to be different, indicating that they cannot be co-located in the same toxin class. A review of the available pectenotoxin toxicity data indicates that the current ARfD that has been set by EFSA needs reviewing, and that an oral dose rate in animal studies of 5000 µg/kg of PTX2 showed no toxicity. Given the foregoing, it is clear that the risk posed by PTXs in shellfish is negligible, risk management controls should be commensurate/relatable to the level of risk posed, and therefore, consideration should be given to the omission of this group from the DSP toxin suite.

## 4. Materials and Methods

### 4.1. Exposure Data

Biotoxin testing performed on commercial and non-commercial samples in New Zealand uses liquid chromatography-tandem mass spectrometry (LC-MS/MS) [4]. Several changes have occurred with the implementation of this method of analysis over the years, with improvements to the technology resulting in improved performance (e.g., limit of detection). Three different tandem quadrupole mass spectrometry systems contributed to the data over the years, a Micromass Quattro Ultima (Manchester, UK) using a Phenomenex Luna C18 150 × 2 mm 5 µm column (Torrance, CA, USA), Micromass Quattro Premier XE (Manchester, UK) using a Phenomenex Luna C18(2) 50 × 1 mm 2.5 µm column (Torrance, CA, USA), and Waters Xevo TQ-S with a Waters Acquity BEH Shield RP18 50 × 2.1 mm 1.7 µm column (Milford, MA, USA). Routinely, a fixed limit of reporting is established, which is reliably able to be achieved by the instrumentation. During the 2009–2019 period, all of the PTX2 results were reported with a reporting limit of 0.01 mg/kg. DSP was calculated as a sum of OA, DTX1, and DTX2 after hydrolysis. Between 2009 and June 2015, the reporting limit for OA, DTX1, and DTX2 was 0.05 mg/kg. It was then reduced to 0.01 mg/kg.

Biotoxin testing and phytoplankton raw data for 2009–2019 were sourced from the Cawthron laboratory information management system (LIMS) database, excluding samples with null entries to either site code or results. For each result, data were exported, including identifiers, site code, site description, sample ID, sample type, sampled date, received date, analysis method, reported name, reported result, and unit. Results for PTX2, PTX2SAs (sum of PTX2SA and 7-epi-PTX2SA), total OA, total DTX1, and total DTX2 were extracted for each sample. DSP was calculated by adding the total OA and DTXs toxins following hydrolysis, i.e., excluding the PTX group. As PTX1, PTX11, and PTX6 were not processed and quantified as part of the monitoring program, no data were available for these congeners to be exported from the LIMS database. Samples from five bloom events were reprocessed to retrospectively quantify PTX1, PTX11, and PTX6, which are acquired in the LC-MS/MS. Raw data for the reprocessed batches (including trace results below the reporting limit) were exported directly from the TargetLynx processing software (Waters Corporation Milford, MA, USA).

For the biotoxin data, data from unclassified site locations, such as overseas product testing (n = 55), imported products (n = 12), and Chatham Island (n = 5) were removed. This yielded a total of 18,947 sample results, with sampling dates spanning 4 January 2009 to 2 September 2019. For phytoplankton data, data from unidentifiable sites (n = 1173) were removed. This yielded a total of 35,277 sample results, with sampling dates spanning 4 January 2009 to 9 September 2019.

Bloom events were classified for shellfish sites within New Zealand from 2009–2019 by first grouping the sites by their sampling zone. Where many samples with overlapping blooms were detected, the zones were separated into subzones by identifying natural barriers which isolate the different regions within the shellfish zones. Blooms were characterized by visually looking at accumulation/depuration patterns in the concentrations over time. Bloom events were assigned if any of the below conditions were observed in at least one sample within the event:

(a) PTX2 was at or above reportable levels (0.01 mg/kg).
(b) DSP toxins were at or above reportable levels (0.05 mg/kg until June 2015, 0.01 mg/kg after June 2015).
(c) PTX2SAs (sum of PTX2SA and 7-epi-PTX2SA) was at or above 0.1 mg/kg (10-fold higher than the reporting limit of 0.01 mg/kg).

The bloom event was determined to start at the first detection of any of the above groups and end at the last detection of any of the above groups. In several cases, if a new bloom had started prior to the previous bloom depurating and the blooms were decided to be far enough apart to be considered as separate events, then the lowest concentration point was used to divide the two events. All samples within the zone or subzone were assigned as part of the bloom event over the time period established. Each bloom event was then reviewed, and any sites that were observed to not have had any toxin detections were excluded.

### 4.2. Risk Assessment

For the deterministic risk assessment, the exposure amount is calculated with the product of the concentration in the meal and the portion size. The exposure was then calculated by dividing the exposure amount by the body weight, assumed to be 60 kg for an adult for comparison against the ARfD. Both the 97.5 percentile and maximum concentrations of PTX2 were used for the exposure calculations with three portion sizes: 100 g, the standard portion size [16]; 268 g, the highest 97.5 percentile portion size of shellfish species for New Zealand consumers; and 400 g, the large portion size adopted by EFSA for risk assessment [6].

For the probabilistic risk assessment, an excel spreadsheet containing PTX2 and DSP data for New Zealand sites/zone and different bivalve species was loaded into the statistical software R 3.6.1. [31] for analysis and the risk characterization simulation. The mc2d package (version 0.1-18) for R was used in the development of the simulation and risk characterization [32].

A similar approach to EFSA [6] was taken for portion sizes, that is, a triangular distribution was used for the portion sizes because insufficient information was available to determine a distribution shape. This distribution was defined by the minimum value of 0 g, most likely value (mode) of 100 g, and maximum value of 400 g. The 400 g large portion is likely an overestimate and hence the likely exposure to PTX2 would also be overestimated.

Two approaches were undertaken to estimate distributions of PTX2 during bloom events only for the exposure modeling: Model 1: A binomial distribution with probabilities of a detection/non-detection that are equal to those in the bloom data set (i.e., 6.55% and 93.45%, respectively). Detects are generated from a log-normal distribution (parameters: meanlog = −4.098, sdlog = 0.445) that was the best fit to the detections, and this was left truncated at the limit of reporting of 0.01 mg/kg. Non-detects are assigned a PTX2 concentration of 0.01 mg/kg, resulting in a conservative, i.e., overestimate of risk. Model 2: Using the empirical distribution of PTX2 concentrations from the bloom data set. Non-detects are assigned a PTX2 concentration of 0.01 mg/kg.

## Figures and Tables

**Table 1:** Summary of the number of samples analyzed, detections, and minimum, maximum, mean, median, and 97.5 percentile (PCTL) concentrations (mg/kg) of PTX2 in different years in New Zealand over the 2009–2019 period.

Year	No. Samples	Detections	% Detected	Min	Max	Mean	Median	97.5 PCTL
2009	1688	56	3.3%	0.010	0.063	0.019	0.015	0.048
2010	1618	14	0.9%	0.010	0.041	0.014	0.011	0.035
2011	1684	21	1.2%	0.010	0.043	0.016	0.014	0.038
2012	1647	13	0.8%	0.011	0.025	0.015	0.013	0.024
2013	1723	5	0.3%	0.010	0.021	0.017	0.019	0.021
2014	1776	10	0.6%	0.010	0.016	0.013	0.014	0.016
2015	1871	66	3.5%	0.010	0.059	0.021	0.017	0.053
2016	1836	21	1.1%	0.010	0.079	0.026	0.021	0.078
2017	1924	14	0.7%	0.010	0.027	0.017	0.017	0.026
2018	1857	12	0.6%	0.011	0.058	0.023	0.017	0.054
2019	1323	19	1.4%	0.010	0.024	0.014	0.012	0.023
Total	18947	251	1.3%	0.010	0.079	0.019	0.015	0.052

**Table 2:** Summary of the number of samples analyzed, detections, and minimum, maximum, mean, median, and 97.5 percentile (PCTL) concentrations (mg/kg) of PTX2 in different months of the year in New Zealand over the 2009–2019 period.

Month	No. Samples	Detections	% Detected	Min	Max	Mean	Median	97.5 PCTL
January	1615	10	0.6%	0.011	0.043	0.020	0.016	0.041
February	1617	30	1.9%	0.010	0.027	0.014	0.012	0.026
March	1679	10	0.6%	0.010	0.023	0.013	0.011	0.021
April	1594	11	0.7%	0.010	0.039	0.016	0.014	0.035
May	1634	10	0.6%	0.011	0.022	0.015	0.015	0.022
June	1574	15	1.0%	0.010	0.058	0.020	0.016	0.052
July	1594	9	0.6%	0.011	0.027	0.016	0.015	0.026
August	1563	21	1.3%	0.010	0.052	0.022	0.018	0.047
September	1514	47	3.1%	0.010	0.059	0.021	0.018	0.054
October	1593	50	3.1%	0.010	0.046	0.017	0.015	0.034
November	1542	28	1.8%	0.010	0.079	0.024	0.017	0.078
December	1428	10	0.7%	0.010	0.063	0.021	0.013	0.058
Total	18947	251	1.3%	0.010	0.079	0.019	0.015	0.052

**Table 3:** Summary of the number of samples analyzed, detections, and minimum, maximum, mean, median, and 97.5 percentile (PCTL) concentrations (mg/kg) of PTX2 in different types of shellfish analyzed in New Zealand over the 2009–2019 period.

Organism ^1^	Sites	No. Samples	Detections	% Detected	Min	Max	Mean	Median	97.5 PCTL
Green-lipped mussel	83	15947	186	1.2%	0.010	0.079	0.019	0.015	0.056
Pacific oyster	22	1141	40	3.5%	0.010	0.027	0.015	0.015	0.026
Clam	11	1042	6	0.6%	0.013	0.027	0.018	0.016	0.026
Scallop	20	298	4	1.3%	0.012	0.032	0.020	0.017	0.031
Dredge oyster	8	228	1	0.4%	0.043	0.043	0.043	0.043	0.043
Surf clam	6	97	5	5.2%	0.010	0.024	0.015	0.012	0.023
Blue mussel	12	56	7	12.5%	0.011	0.042	0.021	0.020	0.039
Queen scallop	2	52	2	3.8%	0.010	0.011	0.011	0.011	0.011
Tuatua	5	28	0						
Pipi	2	19	0						
Cockle	3	17	0						
Oyster	5	9	0						
Abalone	3	8	0						
Geoduck	3	5	0						
Total	144	18947	251	1.3%	0.010	0.079	0.019	0.015	0.052

^1^ Organism as identified in the LIMS database.

**Table 4:** Deterministic intake of PTX2 based on all samples

Parameter	Units	97.5 Percentile	Maximum
Concentration PTX2	mg PTX2/kg	0.01	0.079
Exposure by eating 100 g	µg PTX2/person	1.0	7.9
	µg PTX2/kg b.w.	0.02	0.13
Exposure by eating 268 g	µg PTX2/person	2.7	21.2
	µg PTX2/kg b.w.	0.04	0.35
Exposure by eating 400 g	µg PTX2/person	4.0	31.6
	µg PTX2/kg b.w.	0.07	0.53
